# Equity in COVID-19 Vaccine Resource Distribution: An Exploration of Vaccine Uptake Among Health Workers in a Low-Income Setting

**DOI:** 10.3390/healthcare14040535

**Published:** 2026-02-21

**Authors:** Ifeolu David, Tyler W. Myroniuk, Wilson Majee

**Affiliations:** 1 Department of Population Health Leadership and Analytics, School of Health Professions, University of Texas at Tyler, Tyler, TX 75799, USA; 2 Department of Sociology, University of Kansas, Lawrence, KS 66045, USA; 3 Department of Public Health, College of Health Sciences, University of Missouri, Columbia, MO 65211, USA; majeew@health.missouri.edu

**Keywords:** vaccine uptake, low-income country, health workers, COVID-19, global health

## Abstract

Background: Healthcare workers are at the forefront of the global battle against COVID-19. Their vaccination perspectives, particularly in regions like Sierra Leone that have faced health crises such as the Ebola outbreak, are essential for shaping public health strategies in low-income countries that routinely face infectious disease outbreaks. Objective: This research sought to understand the perceptions and experiences of Sierra Leone’s healthcare workers concerning COVID-19 vaccination and booster doses, set against the backdrop of global health resource disparities and regional vaccine distribution challenges. Methods: Utilizing a mixed-methods approach, the study analyzed data from an online survey, which saw 1001 complete responses from 2060 participants across six Ebola-impacted districts (October–November 2022), and in-depth interviews with 24 health workers from three of these districts (February–July 2022). Results: Approximately 80% of respondents reported having received a COVID-19 vaccine, predominantly Sinopharm and AstraZeneca, yet only 34% of vaccinated participants had received a booster dose. In multivariable analyses, personally knowing someone who experienced serious COVID-19 illness or death was associated with higher odds of both initial vaccination and booster uptake (*p* < 0.05). By contrast, prior Ebola-related experiences were not consistently associated with vaccination outcomes. Qualitative findings contextualized these patterns, highlighting the roles of professional exposure, limited booster-related information, and inequities in vaccine availability and distribution. Conclusion: These findings indicate that vaccination strategies must move beyond initial rollout to address barriers to sustained engagement, particularly for booster uptake among healthcare workers. They also emphasize the need for equitable vaccine access and transparent, locally tailored communication to mitigate structural and informational constraints in low-income settings.

## 1. Introduction

Due to global travel, trade, and climate change, diseases can spread more rapidly than ever before [1,2,3]. The global impact of COVID-19 exemplifies this dynamic, highlighting that health issues in one country can swiftly escalate into concerns for many others [4,5]; geographic disparities in health resource allocation quickly become evident [6].

The significance of immunization is paramount as the world grapples with the lingering effects of the COVID-19 pandemic [7]. Vaccines have historically played a pivotal role in controlling and eradicating infectious diseases, and COVID-19 vaccines are no exception [8]. Yet, as the virus mutates and new variants emerge, the discourse is shifting toward the necessity of updated vaccine doses. Far from being mere supplementary shots, updated COVID-19 doses are integral to global efforts against the virus, ensuring prolonged immunity and adapting to the virus’s evolving nature [9]. However, robust promotion and widespread awareness are crucial for these updated vaccine doses to fulfill their intended purpose, especially on a global scale.

When COVID-19 vaccines were introduced, there was a global urgency to distribute them, which inadvertently led to enormous disparities in access [10,11,12]. These disparities were especially pronounced in low-income settings, where socioeconomic and infrastructural challenges act as significant barriers to obtaining timely access to the vaccines, as emphasized by Acharya, Ghimire [13]. The positioning of these regions within the global supply chain further exacerbated the issue, generating a perception of secondary importance among individuals in these areas. Such circumstances necessitate a nuanced understanding, as it intensifies distrust and hesitancy. As a result, when vaccines finally reach the least prioritized locations, they are often received with skepticism, rooted in these multifaceted factors [14].

Historical patterns reveal pronounced disparities in global health resource distribution during public health emergencies. For instance, during the HIV/AIDS epidemic’s peak, wealthier nations often accessed treatments and preventive measures promptly, leaving low-income countries grappling with scarce resources [15,16]. Despite advancements in HIV management options, the adoption of prevention strategies such as Pre-Exposure Prophylaxis (PrEP) remains notably limited in low-income settings [17]. The Ebola outbreak followed a similar trajectory, with countries like Sierra Leone, Liberia, and Guinea witnessing over 11,000 deaths, while most high-income countries maintained case counts below ten [18]. The Ebola outbreak also highlighted the delay in global support for affected regions, with sustained coordinated assistance only materializing months after the outbreak’s onset [19]. Such disparities go beyond mere logistical obstacles and carry profound ethical and socioeconomic implications globally. In an era marked by medical advancements capable of preventing and treating numerous diseases, the unequal distribution of disease prevention breakthroughs can lead to avoidable deaths and extended health crises in low-resourced settings.

In response to disparities in health resources, global health entities like the World Health Organization (WHO) and the Global Alliance for Vaccines and Immunization, have launched programs targeting equitable vaccine and treatment distribution. The COVAX initiative, for instance, aimed to ensure that COVID-19 vaccines are made accessible to low- and middle-income countries, promoting a more balanced distribution [20]. Similarly, the Global Fund to Fight AIDS, Tuberculosis, and Malaria has been pivotal in directing resources to low-income regions to combat infectious diseases [21]. While these initiatives signify a move towards a more equitable global health strategy, there is substantial work ahead. The effectiveness of these initiatives, especially in the context of COVID-19, remains to be fully assessed, given the persistently low vaccine uptake rates in regions like sub-Saharan Africa.

Consistent with the Health Belief Model, vaccination behavior has been shaped by perceived susceptibility to disease, perceived severity of outcomes, perceived benefits of vaccination, perceived barriers, and salient cues to action [22,23,24]. Prior epidemic exposure—such as the Ebola outbreak in Sierra Leone—may function as a lasting cue to action that heightens perceived risk while also shaping how individuals evaluate the benefits and constraints of subsequent vaccination efforts [25].

Drawing from evidence among healthcare workers in Sierra Leone, a low-income country (LIC) setting shaped by repeated infectious disease outbreaks, this paper examines how global COVID-19 vaccination strategies translated into local availability and uptake. Healthcare workers occupy a distinctive position in vaccination decision-making because professional exposure heightens perceived vulnerability to infectious disease, while direct engagement with health systems sharpens awareness of both vaccine benefits and structural constraints. We therefore anticipated high initial vaccine uptake among clinical staff whose routine patient contact amplifies risk appraisal. We further hypothesized that prior Ebola-related experiences would be associated with COVID-19 vaccination behavior, and we examined whether these associations varied across stages of vaccine engagement, including initial vaccination and booster uptake.

## 2. Methods

### 2.1. Study Design

Our data come from a comprehensive mixed-methods [26] study conducted between February 2022 and October 2022 across six districts in Sierra Leone: Freetown, Kenema, Makeni, Port Loko, Bo, and Magburaka [27]. These districts recorded some of the highest caseloads during both the 2014–2016 Ebola outbreak and the COVID-19 pandemic and collectively account for a substantial proportion of the national healthcare workforce. The study was implemented in two phases designed to elicit complementary perspectives on COVID-19 vaccine and booster uptake. Because no comprehensive national sampling frame of healthcare workers exists in Sierra Leone, probability-based sampling was not feasible. We therefore employed non-probability sampling strategies appropriate for low-resource and emergency contexts [28,29]. Eligibility criteria for participation in both phases included being aged 18 years or older and holding a healthcare role within the selected districts. While the resulting sample is not statistically representative of all healthcare workers nationally, the purposive selection of high-burden districts corresponds to regions that account for a substantial share of the national health workforce [30,31]. The sample, therefore, reflects workforce concentrations in settings most affected by epidemic strain and is analytically informative for understanding vaccination experiences in these contexts.

In the first phase, an exploratory qualitative approach [32] was adopted, with in-depth interviews conducted between February and July 2022. Purposive sampling with snowball techniques was used to recruit healthcare workers (N = 24). Interviews lasted between 40 and 80 min and were conducted in English or Krio, depending on participant preference. All participants provided informed consent, and interviews were audio-recorded for accuracy. Findings from the qualitative phase informed the development of the survey instrument used in the second phase.

The second phase involved an online survey administered via REDCap [33] between October and November 2022. The survey was distributed through administrators of health facilities and professional health worker groups on WhatsApp and other social media platforms across the six study districts. The survey required approximately 10–15 min to complete and included items on demographics and perceptions of COVID-19 and vaccination. Survey engagement was defined as accessing the survey link and responding to at least one item. A total of 2060 respondents engaged with the survey, of whom 1001 completed all survey items and were therefore included in the final analytic sample. Additional methodological details describing the study design and data collection procedures have been reported previously [27].

### 2.2. Analysis

#### 2.2.1. Quantitative

For quantitative analyses (which are presented first in this paper), descriptive statistics, chi-square tests (bivariate inferences) [34], and logistic regression (multivariate inferences) were utilized. Our two outcomes of interest were: (a) having received a COVID-19 vaccination; and (b) having received a COVID-19 booster, conditional on prior vaccination. Given the binary nature of the outcome variables, logistic regression was used to assess how prior Ebola experiences and COVID-19 mortality were associated with COVID-19 vaccination and booster uptake, alongside key individual and regional characteristics [35]. Key outbreak-related independent variables were added sequentially into models and then included simultaneously in a final model to assess how each variable was associated with vaccination outcomes when considered independently and when estimated together with the other outbreak-related variables. Separate logistic regression models were estimated for each outcome: (1) having received a COVID-19 vaccine (1 = yes; 0 = no), and (2) having received a COVID-19 booster following vaccination (1 = yes; 0 = no). Each model included an intercept term and three sets of explanatory variables. First, individual-level control variables were included: whether the respondent provides direct medical care to patients, length of time as a healthcare worker, educational attainment, prior COVID-19 diagnosis, age, and sex assigned at birth. Second, region fixed effects were included to account for district-level characteristics corresponding to where healthcare workers were working at the time of the survey, thereby adjusting for geographic heterogeneity in health system context and epidemic exposure. Finally, the key outbreak-related independent variables were included: (a) prior infection with the Ebola virus (1 = yes; 0 = no); (b) having a colleague, friend, and/or family member who died from Ebola (1 = yes; 0 = no); and (c) personally knowing someone who experienced serious illness or died from COVID-19 (1 = yes; 0 = no). Model parameters were estimated using maximum likelihood procedures, and standard assumptions for logistic regression were applied.

#### 2.2.2. Qualitative

Utilizing thematic analysis as outlined by Guest, MacQueen [36], the qualitative data were analyzed using a systematic coding, and interpretation approach. The initial six interviews were analyzed to identify common themes and unique insights, which then informed the interview prompts for the subsequent 18 interviews. Interviews continued until thematic saturation was reached, defined as the point at which no substantively new themes or dimensions emerged in successive interviews [37]. Saturation was assessed iteratively during data collection and analysis, and was determined to have been achieved within the final interviews. Based on the themes identified, a preliminary codebook was developed and tested on three randomly chosen transcripts. Once validated, the codebook was applied to the remaining transcripts using the Atlas.ti software version 25. This paper specifically zooms in on two primary themes: “COVID-19 vaccine uptake” and “COVID-19 vaccine booster uptake”. To delve deeper, an additional coding round was conducted, resulting in the identification of pertinent sub-themes detailed in the results section. While findings are presented with participant pseudonyms and roles, no significant variations were observed across these categories.

**Figure 1:**
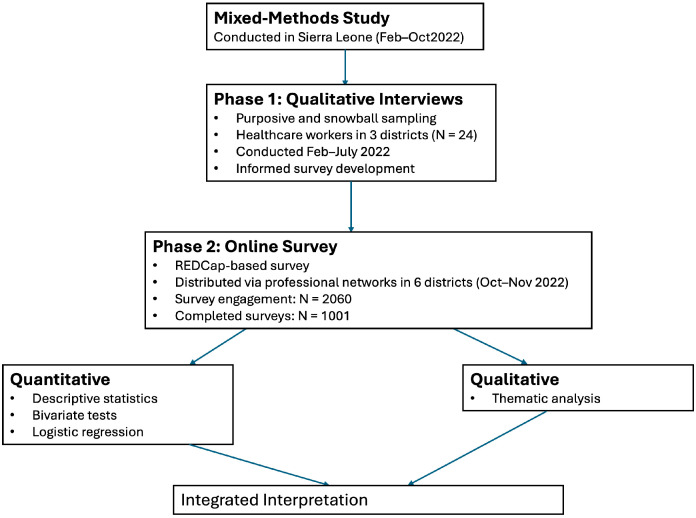
Summary of study procedures.

We present the analyses of these data collection modalities separately in the results section, below, and then triangulate the results in Section 5. See Figure 1 for a summary of the study procedures.

## 3. Quantitative Findings

### 3.1. Descriptive Statistics

A total of 81% of participants who completed the online survey reported having been vaccinated against COVID-19. The most common vaccines received were Sinopharm and AstraZeneca, accounting for 51% of the vaccinated participants. The primary motivations for getting vaccinated were personal protection (69%), safeguarding family and friends (51.9%), and protecting co-workers (33.6%). Concerns about potential side effects of the vaccine, which might prevent them from working, emerged as the predominant barrier to vaccination among the unvaccinated (47%). Interestingly, this concern was also cited by 61% of vaccinated individuals as a significant challenge they had to overcome before deciding to get vaccinated. While the primary COVID-19 vaccine uptake among health workers was moderately high, only 34% of the vaccinated individuals had received a booster vaccine dose. The predominant reasons for not receiving a booster dose were a lack of awareness (59%) and uncertainty about the necessity of a booster dose (14%). Additional descriptive statistics on demographic variables can be found in Table 1 below. Additional descriptive statistics regarding vaccine characteristics, motivation, and barriers are presented in Supplementary Table S3.

### 3.2. Inferential Analyses

Bivariate analyses indicated that COVID-19 vaccination uptake was associated with outbreak-related experiences. Vaccination was more common among respondents who reported Ebola infection (χ^2^; *p* = 0.001) or Ebola-related mortality within their social or professional networks (χ^2^; *p* = 0.001), and among those who personally knew someone who experienced severe COVID-19 illness or death (χ^2^; *p* = 0.000). In contrast, respondents’ own prior Ebola infection was not associated with COVID-19 vaccination status.

Logistic regression analyses estimating the log odds of COVID-19 vaccination (found in Table 2, below, and in Supplementary Table S1) showed a more nuanced pattern. After accounting for individual, occupational and regional controls, only one association found in the bivariate analyses persisted: personally knowing someone who experienced serious illness or died from COVID-19 was associated with higher log odds of respondents being vaccinated (*p* = 0.026, Model 3; *p* = 0.024, Model 4). However, respondents who had been infected with the Ebola virus were less likely to have been vaccinated against COVID-19 (*p* = 0.077, model 1; *p* = 0.007, model 4); the more pronounced coefficient in Model 4 suggests that Ebola- and COVID-19–related experiences capture overlapping experiential pathways, such that their simultaneous inclusion attenuates associations observed in unadjusted models. These differences indicate that some bivariate associations reflected unadjusted relationships that were attenuated after accounting for individual, occupational, and regional characteristics, as well as overlapping outbreak-related experiences.

Among respondents who were initially vaccinated against COVID-19, prior Ebola and COVID-19 experiences appeared to influence decisions to receive a COVID-19 booster. Bivariate analyses indicate that getting a booster dose was associated with knowing someone in one’s family, friends, or community network who had experienced serious COVID-19 illness or death (χ^2^; *p* = 0.033). Approximately 13% more respondents received a booster if previously infected with Ebola (χ^2^; *p* = 0.021). These findings suggest that personal experience with Ebola may have had a lasting impact on healthcare workers’ attitudes towards vaccination, translating into higher booster dose uptake.

Logistic regression analyses (found in Table 3, below, and Supplementary Table S2) temper this pattern after adjustment for demographic and personal characteristics, and region fixed effects. Similar to findings related to the initial COVID-19 vaccination, personally knowing someone who experienced serious illness or died from COVID-19 was associated with higher log odds of respondents receiving a booster (*p* = 0.012, model 3; *p* = 0.035, model 4). However, when prior COVID-19 and Ebola-related experiences were simultaneously included in the model (Model 4), neither prior Ebola infection nor knowing a colleague, friend, or family member who died from Ebola was significantly associated with COVID-19 booster uptake, compared with respondents without these Ebola-related experiences.

Bivariate and multivariate analyses show a consistent association between COVID-19 experiences and subsequent vaccination with initial and booster COVID-19 vaccine doses.

## 4. Qualitative Findings

Out of 24 interview participants, 21 had been vaccinated for COVID-19. This uptake rate aligns with the findings from our online survey. However, both vaccinated and unvaccinated participants voiced concerns regarding the COVID-19 vaccines available in Sierra Leone and their distribution. These concerns also influenced the uptake of the booster doses as participants were either not aware or did not have enough information to know that it was needed. The results are organized into two main themes: Vaccine Uptake and Booster Vaccine Uptake.

### 4.1. Vaccine Initial Dose Uptake

#### 4.1.1. Vaccine Concerns

A recurring sentiment was the disparity in vaccine options available in Sierra Leone compared to high-income countries like the USA. The early vaccine rollout in Sierra Leone primarily offered Sinopharm and AstraZeneca. Participants perceived these as less preferable options, especially when aware of the differing vaccine choices available in countries like the USA. This notion was expressed by a participant as follows *“Well, I wasn’t a fan of a Chinese-made vaccine and the conspiracy theories surrounding it”—Dr. Peter, male MD*.

There was also a perception that the AstraZeneca doses shipped to Sierra Leone from the UK might be less viable and potentially unsafe. One female nurse argued that comparing the safety of vaccines in Sierra Leone to those in the USA was not entirely valid due to these differences *“We can’t compare ourselves with the USA”—Cecilia, female nurse.*

Stories of adverse reactions after vaccination also contributed to increased hesitancy. For instance, a colleague of one of the participants fell ill after receiving a vaccine, leading to heightened concerns among her peers *“I had a friend who knew someone who had severe symptoms following the vaccine uptake, that individual got really sick and well yes, it was scary for me.”—Phebian, female nurse.*

Collectively, these concerns suggest that vaccine hesitancy among study participants was shaped by perceived inequities in vaccine quality, rather than opposition to vaccination itself. These perceptions reflect broader global health inequities in vaccine allocation and transparency, reinforcing how structural disparities, rather than generalized hesitancy, shaped early vaccination deliberations in this low-income setting.

#### 4.1.2. Vaccine Information and Distribution Concerns

A significant challenge was the perceived lack of evidence-based information on the vaccines available in Sierra Leone. This uncertainty was particularly pronounced for the Sinopharm vaccine as described by another participant: *“Currently, there isn’t enough evidence to show that the vaccines are effective”—Georgia, female public health staff*.

Conspiracy theories further exacerbated these concerns, with some participants believing that vaccines distributed to Africa had ulterior motives.

There was a lot of conspiracy theories around the vaccine, especially during the early rollout. Some believed the vaccines distributed to Africa were to reduce fertility or could lead to death.

—*Cecilia*

The mode of vaccine distribution also raised concerns among participants. Individuals felt they had little agency in choosing their vaccine. Instead, the vaccination team made decisions based on reported underlying health conditions.

There were two options. Those with medical conditions and the elderly were given AstraZeneca. Others, young and without underlying conditions, received Sinopharm. I didn’t have an option; I was given what was recommended.

—*Abdul, male nurse*

The rationale behind this distribution method was unclear to many, including medical doctors.

I didn’t get to choose. They had Sinopharm and AstraZeneca. The choice of vaccine was determined by the vaccinators based on their criteria. They never really explained why.

—*Dr. Alusine, male MD*

These accounts suggest that limited transparency in vaccine allocation and insufficient communication about vaccine efficacy constrained healthcare workers’ sense of agency, undermining confidence even among medically trained professionals. Although such concerns were widespread, they did not ultimately prevent high initial vaccine uptake, suggesting that perceived COVID-19 risk may have outweighed these structural reservations. This interpretation aligns with the quantitative finding that personal connection to severe COVID-19 illness remained the most consistent correlate of COVID-19 vaccination.

### 4.2. Booster Vaccine Uptake

#### 4.2.1. Lack of Awareness and Limited Promotion

Despite the global emphasis on the importance of booster doses the concept of a booster dose was unfamiliar to many participants. Even those who had received their initial doses were largely uninformed about the need for subsequent booster doses. This was evident by remarks from a female nurse when asked about the booster vaccine dose *“We haven’t heard about a third dose at all. It’s just the two doses which I’ve already got.”—Christiana*. Several participants echoed this sentiment, emphasizing the lack of communication regarding the booster dose.

A few participants who had some knowledge about the booster doses felt that the promotion and sensitization around it were inadequate. They believed the communication around booster COVID-19 vaccine doses was not as robust as that of the initial vaccine rollout.

I’ve heard about the booster vaccine dose, but the promotion has been vague. It’s nothing compared to the original vaccine sensitization. I didn’t see stakeholders, like the president, taking the booster like when the initial vaccine was introduced.

—*Abass, male nurse*.

Another participant, a medical doctor, expressed his willingness to receive a booster dose but highlighted the lack of information as a barrier; *“Even though I wasn’t told to get the booster, I believed I would need one. However, there’s no information or sensitization about it.”*—Dr. Alusine

The absence of clear communication seemed to have deterred many health workers from seeking out and receiving the booster dose. Hence, the promotion of COVID-19 vaccine booster doses was perceived as lacking, especially when compared to the initial vaccine rollout. The contrast between strong initial vaccine sensitization and minimal booster communication highlights how institutional signaling influenced perceptions of vaccine necessity, leading to reduced engagement after the emergency framing of COVID-19 faded.

#### 4.2.2. Personal Health Concerns and Complacency

Health concerns, especially reactions to the initial doses of the COVID-19 vaccine, made some participants cautious about receiving booster doses. When asked how many doses of the vaccine she believes she would need, a female nurse replied as follows;

They said up to three doses, but I only took one. I was scheduled for the second dose but didn’t go for it. After feeling unwell from the first dose and being on medications that reduce the immune system, I became hesitant.

—*Susan*

A sense of complacency was also evident among some participants. They believed that the initial doses provided sufficient protection, especially given the mild nature of COVID-19 infections in Sierra Leone.

I’ve got the first and second doses but haven’t gone for the third dose yet. I think the pandemic has waned, and the doses I’ve received have given me immunity. The COVID-19 infection is very mild for us here in Sierra Leone, especially after vaccination. I’m also young with no comorbid condition, so I don’t see the need for another dose.

—*Dr. John, male MD*

These narratives demonstrate that prior vaccination experiences and perceptions of reduced local COVID-19 severity influenced the perceived need for continued vaccination, particularly in the absence of sustained institutional guidance. Overall, booster disengagement was not driven by ideological resistance but by structural and informational constraints common to resource-limited settings. Limited institutional signaling and inconsistent health communication reflect broader challenges in vaccine governance, helping to explain the decline in booster uptake despite generally positive attitudes toward vaccination and reinforcing the importance of perceived COVID-19 risk in shaping vaccination decisions among this population.

## 5. Discussion

The findings of this mixed-method study provide valuable insights into the perspectives and experiences of healthcare workers in Sierra Leone regarding COVID-19 vaccination. The high uptake of the COVID-19 vaccine among participants, despite widespread concerns narrated in the in-depth interviews, is a testament to the resilience and lessons learned following the massive Ebola outbreak in the region in 2014. This commendable uptake aligns with global efforts to achieve herd immunity and suggests robust support for the science of vaccination among healthcare professionals in Sierra Leone. The vaccine uptake rates among the study population are also similar to those from studies conducted in high-income settings such as the USA and significantly surpass that of other low-income countries in sub-Saharan Africa [38,39,40]. The findings suggest that experiential factors informed early vaccination decisions, while subsequent engagement was influenced more decisively by structural conditions around vaccine access, distribution, and communication. Hence, although booster uptake was low in the quantitative study, in-depth interviews revealed substantial willingness to receive a booster, indicating that limited awareness and communication, rather than reluctance, contributed to low coverage. The barriers identified in the findings are therefore substantial, as the uptake rates of booster doses lag significantly behind those of health workers in high-income settings [41,42,43]. Overall, direct personal connection to severe COVID-19 illness or death was the most consistent factor associated with both initial vaccination and booster uptake. Qualitative findings reinforce this pattern by demonstrating that healthcare workers’ vaccination decisions were influenced by perceived COVID-19 risk, while uptake of subsequent doses declined as personal exposure became less salient and institutional signaling weakened. Consistent with studies from other regions, our findings suggest that perceived COVID-19 risk is a key correlate of vaccination behavior [44,45,46,47,48].

Despite the positive trends in vaccine uptake, the study highlights two major concerns that could potentially hinder global efforts towards pandemic recovery. Firstly, the disparity in vaccine options between Sierra Leone and high-income countries, such as the USA where Pfizer and Moderna are common, underscores the broader global issue of vaccine equity [49]. As depicted in participant narratives from the in-depth interviews, promoting vaccination in LICs in the absence of these vaccine options opens huge opportunities for doubts and mistrust among a population whose history has been marred by trans-Atlantic slavery, colonization, and exploitation of mineral resources by high-income countries [50]. Hence, the perception that vaccines available in the region might be of a different quality and can undermine trust in the vaccination process. Addressing these concerns requires transparent communication about the efficacy and safety of all vaccines and ensuring global accessibility. Secondly, the unfamiliarity with the concept of a booster dose among many participants in both quantitative and qualitative studies is concerning. This knowledge gap emphasizes the need for continuous education and communication, even after the initial vaccine rollout. Numerous questions have emerged concerning the suboptimal rates of COVID-19 vaccine booster uptake in low-resource settings, particularly in sub-Saharan Africa, leaving many aspects more ambiguous than clarified [51]. This paper takes a novel approach by highlighting the role of limited health promotion in the region. The study findings therefore highlight the importance of public health promotion efforts in the region for global pandemic recovery.

The findings from this study have important implications for global vaccine distribution mechanisms, including the COVID-19 Vaccines Global Access (COVAX) initiative [20]. While COVAX played a critical role in facilitating initial vaccine access in Sierra Leone, including the delivery of vaccines such as Sinopharm and AstraZeneca through multilateral and bilateral partnerships [52], our results suggest that initial access alone is insufficient to sustain vaccine engagement over time. The pronounced drop in booster uptake observed in this study highlights structural limitations in global distribution frameworks that prioritize primary dose coverage but provide less consistent support for booster availability, updated formulations, and downstream health communication. As global vaccine strategies evolve, mechanisms such as COVAX may need to expand beyond emergency supply functions to incorporate sustained delivery, clearer guidance on booster regimens, and closer coordination with national health systems to support continued vaccine engagement in low-income settings.

Beyond the design of specific distribution mechanisms such as COVAX, these findings also underscore broader structural inequities in global vaccine allocation. The disparities in health resource allocation are starkly evident in the distribution of the COVID-19 vaccines. While high-income settings, such as the USA, are making continuous efforts towards vaccination and even implementing updated vaccine doses [9], many in LICs, particularly in sub-Saharan Africa, still lack access to their initial doses of preferred vaccine products. These regions not only fall short of baseline vaccination goals but also have limited knowledge about the importance of booster doses and updated vaccine regimens. This selective support for COVID-19 vaccine regimens could intensify health disparities, making certain regions perpetually vulnerable. Compounding the concern is the emergence of new COVID-19 variants, against which previous vaccine formulations may prove less effective [53]. With the recent CDC-recommended updated booster vaccine doses completely unavailable in LICs like Sierra Leone, promoting the use of additional doses of COVID-19 vaccines represented as boosters may not have the desired effect in preventing the spread of new strains of the COVID-19 virus. This becomes even more relevant as evidence from the CDC suggests that recent cases of COVID-19 in the USA and globally have been predominantly (86%) due to the more recent JN.1 strain of SARS-CoV-2 [54]. Hence, the current global approach to COVID-19 vaccination could prolong the duration of the pandemic recovery and also increase the risk of new variants emerging from regions where many people are not fully vaccinated or haven’t received booster vaccine doses. More significantly, the updated versions of the Pfizer and Moderna COVID-19 vaccines, recommended by the CDC, may encounter considerable delays in being accessible to LICs. These issues are particularly relevant given the current focus on global recovery from the pandemic and the push for unified health strategies [55]. Both economic and social outcomes could be significantly impacted by these trends, emphasizing that equity in vaccine resource distribution is paramount.

### Study Strengths and Limitations

This study has several valuable strengths that enhance the interpretation of its findings. By integrating quantitative and qualitative data, the analysis provides insight into factors shaping both initial COVID-19 vaccination and subsequent booster uptake among healthcare workers. Although the sample is not nationally representative, it is analytically informative for understanding COVID-19 vaccination experiences in regions heavily affected by prior Ebola outbreaks. The study offers a rare empirical account of healthcare worker perspectives in a low-resource setting with recent epidemic experience, contributing valuable evidence on how prior outbreak exposure and structural conditions intersect to shape vaccination behavior.

At the same time, the findings should be interpreted in light of several limitations. The use of non-probability sampling limits the representativeness of the sample, and the results cannot be generalized to all healthcare workers in Sierra Leone. In addition, vaccination decisions are likely influenced by unobserved or unmeasured characteristics, such as broader health histories, early life experiences, and personal dispositions, which could not be fully captured through the survey and interview data. Finally, the cross-sectional design and reliance on retrospective self-reports of vaccination and prior health events preclude causal inference regarding the relationship between outbreak experiences and vaccination decisions, and introduce the potential for recall bias and measurement error.

## 6. Conclusions

The findings of this study suggest that global public health support should be conceptualized not as discretionary assistance from high-income countries, but as an integral component of global health security. Among healthcare workers in Sierra Leone, initial COVID-19 vaccination uptake was relatively high, whereas booster uptake was substantially lower, suggesting that determinants of vaccination behavior vary across stages of vaccine engagement. Quantitative results demonstrate that direct personal connection to severe COVID-19 illness or death was more consistently associated with vaccination outcomes than prior Ebola-related experiences, while qualitative findings indicate that sustained engagement with vaccination was influenced primarily by structural and informational constraints rather than individual-level hesitancy.

These findings illustrate how global health inequities operate beyond vaccine availability alone, extending to communication, follow-up, and the continuity of vaccination efforts. As the emergence of new viral variants continues to challenge pandemic recovery, maintaining vaccination coverage and uptake of updated COVID-19 vaccine doses remains a critical public health objective. Addressing disparities in health resource distribution and strengthening transparent, ongoing communication are therefore essential for sustaining vaccination programs and enhancing preparedness for future infectious disease outbreaks in an increasingly interconnected global context.

## Figures and Tables

**Table 1:** Demographic characteristics of participants.

	Observations	%
**Education**		
Less than college	249	25.2
At least college	741	74.8
Total	990	100.0
**Sex**		
Female	469	46.9
Male	531	53.1
Total	1,000	100.0
**Age**		
18–25	268	26.8
26–33	350	35.0
34–40	209	20.9
Above 40	174	17.4
Total	1001	100.0
**Region of work**		
Freetown	377	38.7
Makeni	171	17.5
Kenema	75	7.7
Port Loko	103	10.6
Bo	111	11.4
Magburaka	49	5.0
Other	89	9.1
Total	975	100.0
**Years of experience**		
Less than 1 year	208	23.7
1 to 3 years	289	32.9
4 to 5 years	153	17.4
6 to 10 years	150	17.1
More than 10 years	79	9.0
Total	879	100.0
**Provides direct medical care to patients**		
No	331	35.0
Yes	616	65.0
Total	947	100.0
**Prior COVID-19 diagnosis**		
No	849	85.2
Yes	148	14.8
Total	997	100.0
**Prior Ebola Diagnosis**		
No	898	90.2
Yes	98	9.8
Total	996	100.0
**Colleagues, Friends, and/or Family Died from Ebola**		
No	490	50.2
Yes	486	49.8
Total	976	100.0
**Personally knowing someone with severe COVID-19 illness/death**		
No	619	62.3
Yes	374	37.7
Total	993	100.0

**Table 2:** Estimating the Log Odds of COVID-19 Vaccination.

	(1)	(2)	(3)	(4)
Was infected with the Ebola virus (ref. was not)	−0.57 *p* = 0.077			−0.91 *p* = 0.007
Colleague, friend, and/or family member died from Ebola		0.27 *p* = 0.193		0.37 *p* = 0.104
Personally know someone who experienced serious illness or died from COVID-19			0.50 *p* = 0.026	0.54 *p* = 0.024
Observations	847	842	848	836
Pseudo *R*^2^	0.106	0.106	0.115	0.127

Note: Two-tailed tests. Continuous *p*-values presented. Control variables included in the model but not presented here are: whether the respondent provides direct medical care to patients, length of time as a health care worker, education, prior COVID-19 infection, age, sex at birth, work region fixed effects, and the model constant. A full set of coefficients for these models can be found in the Supplementary Table S1.

**Table 3:** Estimating the Log Odds of COVID-19 Booster Vaccination.

	(1)	(2)	(3)	(4)
Was infected with the Ebola virus (ref. was not)	0.28 *p* = 0.196			0.04 *p* = 0.882
Colleague, friend, and/or family member died from Ebola		0.39 *p* = 0.029		0.29 *p* = 0.132
Personally know someone who experienced serious illness or died from COVID-19			0.45 *p* = 0.012	0.39 *p* = 0.035
Observations	703	698	705	696
Pseudo *R*^2^	0.099	0.099	0.104	0.103

Note: Two-tailed tests. Continuous *p*-values presented. Control variables included in the model but not presented here are as follows: whether the respondent provides direct medical care to patients, length of time as a health care worker, education, prior COVID-19 infection, age, sex at birth, work region fixed effects, and the model constant. A full set of coefficients for these models can be found in the Supplementary Table S2.

## Data Availability

The data that support the findings of this study are not publicly available because of privacy and ethical restrictions. De-identified data may be obtained from the corresponding author upon reasonable request and completion of a data-sharing agreement.

## Supplementary Materials

The following supporting information can be downloaded at: https://www.mdpi.com/article/10.3390/healthcare14040535/s1, Table S1: Estimating the Log Odds of COVID-19 Vaccination; Table S2: Estimating the Log Odds of COVID-19 Booster Vaccination, Table S3: Vaccine Characteristics, Motivations, and Barriers.
