# Supplementary material for: Equity in COVID-19 Vaccine Resource Distribution: An Exploration of Vaccine Uptake Among Health Workers in a Low-Income Setting
Source: Healthcare (Basel). 2026 Feb 21;14(4):535. doi: 10.3390/healthcare14040535 (PMC12941062; doi:10.3390/healthcare14040535)
Supplement: Supplementary file 1 [file healthcare-14-00535-s001.zip › healthcare-4051151-supplementary.pdf]

Supplementary Table S1: Estimating the Log Odds of COVID-19 Vaccination

	(1)	(2)	(3)	(4)
Was infected with the Ebola virus (ref. was not)	-0.57<br>p=0.077			-0.91<br>p=0.007
Colleague, friend, and/or family member died from Ebola		0.27<br>p=0.193		0.37<br>p=0.104
Personally know someone who experienced serious illness or died from COVID-19			0.50<br>p=0.026	0.54<br>p=0.024
Currently provides direct medical care to patients (ref. does not)	0.95<br>p=0.000	0.99<br>p=0.000	0.93<br>p=0.000	0.89<br>p=0.000
Length of time as a health care worker? (ref. less than 1 year)				
1 to 3 years	0.06<br>p=0.819	0.06<br>p=0.826	0.07<br>p=0.780	-0.01<br>p=0.966
4 to 5 years	0.25<br>p=0.456	0.22<br>p=0.514	0.29<br>p=0.401	0.27<br>p=0.432
6 to 10 years	0.71<br>p=0.064	0.71<br>p=0.067	0.75<br>p=0.057	0.61<br>p=0.129
More than 10 years	1.16<br>p=0.031	1.13<br>p=0.038	1.08<br>p=0.044	0.95<br>p=0.083
Completed at least some college (ref. did not)	0.31<br>p=0.202	0.32<br>p=0.177	0.35<br>p=0.146	0.39<br>p=0.110
Was previously diagnosed with COVID-19 (ref. no)	0.25<br>p=0.423	-0.06<br>p=0.839	0.02<br>0.959	0.00<br>p=0.994
Age (integer)	-0.00<br>p=0.825	-0.01<br>p=0.688	-0.00<br>p=0.737	0.00<br>p=0.985
Male sex assigned at birth, on original birth certificate (ref. female)	0.39<br>p=0.054	0.35<br>p=0.082	0.37<br>p=0.068	0.38<br>p=0.068
Work Region Fixed Effects Included?	Yes	Yes	Yes	Yes
Constant	0.08<br>p=0.857	-0.02<br>p=0.965	-0.10<br>p=0.821	-0.32<br>p=0.487
Observations	847	842	848	836
Pseudo R^2	0.106	0.106	0.115	0.127

Note: Two-tailed tests. Continuous p-values presented.

Supplementary Table S2: Estimating the Log Odds of COVID-19 Booster Vaccination

	(1)	(2)	(3)	(4)
Was infected with the Ebola virus (ref. was not)	0.28<br>p=0.307			0.04<br>p=0.882
Colleague, friend, and/or family member died from Ebola		0.39<br>p=0.029		0.29<br>p=0.132
Personally know someone who experienced serious illness or died from COVID-19			0.45<br>p=0.012	0.39<br>p=0.035
Currently provides direct medical care to patients (ref. does not)	0.27<br>p=0.196	0.27<br>p=0.199	0.24<br>p=0.257	0.24<br>p=0.254
Length of time as a health care worker? (ref. less than 1 year)				
1 to 3 years	-0.42<br>p=0.073	-0.45<br>p=0.058	-0.42<br>p=0.075	-0.44<br>p=0.064
4 to 5 years	-0.66*<br>p=0.022	-0.70<br>p=0.015	-0.69<br>p=0.018	-0.72<br>p=0.014
6 to 10 years	-1.49<br>p=0.000	-1.59<br>p=0.000	-1.55<br>p=0.000	-1.63<br>p=0.000
More than 10 years	-1.69<br>p=0.000	-1.89<br>p=0.000	-1.77<br>p=0.000	-1.91<br>p=0.000
Completed at least some college (ref. did not)	-0.51<br>p=0.018	-0.44<br>p=0.046	-0.52<br>p=0.017	-0.44<br>p=0.045
Was previously diagnosed with COVID-19 (ref. no)	0.71<br>p=0.003	0.66<br>p=0.005	0.68<br>p=0.004	0.60<br>p=0.014
Age (integer)	0.01<br>p=0.244	0.02<br>p=0.173	0.02<br>p=0.139	0.02<br>p=0.134
Male sex assigned at birth, on original birth certificate (ref. female)	-0.42<br>p=0.016	-0.41<br>p=0.021	-0.44<br>p=0.013	-0.41<br>p=0.020
Work Region Fixed Effects Included?	Yes	Yes	Yes	Yes
Constant	-0.52<br>p=0.204	-0.80<br>0.060	-0.75<br>p=0.072	-0.95<br>p=0.031
Observations	703	698	705	696
Pseudo R^2	0.099	0.099	0.104	0.103

Note: Two-tailed tests. Continuous p-values presented.

Supplementary Table S3. Vaccine Characteristics, Motivations, and Barriers

**Ever received a COVID vaccine?**		
No	185	18.8
Yes	801	81.2
Total	986	100.0
**Vaccine product received**		
Sinopharm	162	20.2
AstraZeneca	249	31.1
Jansen (Johnson& Johnson)	151	18.9
Pfizer	31	3.9
Moderna	26	3.2
I don't know	155	19.4
I prefer not to respond	27	3.4
Total	801	100.0
***Motivations for vaccination (N=801)**		
Self-protection	555	69.3
Protecting family and friends	416	51.9
Protecting co-workers	269	33.6
Protecting community	300	37.5
***Barriers to vaccination among vaccinated (N=801)**		
Concerns about side effect preventing work	490	61.2
No Barrier	185	23.1
***Barriers to vaccination among unvaccinated (N=185)**		
Concerns about side effect preventing work	87	47.0
Dislike for the vaccine	15	8.1
Distrust for Western countries	11	5.9
****Ever received a COVID-19 booster vaccine dose**		
No	511	65.2
Yes	273	34.8
Total	784	100.0
****Reasons for not getting a booster dose**		
Not aware	249	49.3
I thought I was fully vaccinated	48	9.5
Not sure I meet the requirement	25	5.0
I don't think I need a booster	44	8.7
Other	139	27.5
Total	505	100.0

*Select all that apply; percentages are calculated using the number of respondents who answered each specific question as the denominator and may therefore exceed 100%.

**A subset of participants who had received the COVID-19 vaccine.
